# Computerized Analysis of Mammogram Images for Early Detection of Breast Cancer

**DOI:** 10.3390/healthcare10050801

**Published:** 2022-04-25

**Authors:** Yassir Edrees Almalki, Toufique Ahmed Soomro, Muhammad Irfan, Sharifa Khalid Alduraibi, Ahmed Ali

**Affiliations:** 1 Department of Medicine, Division of Radiology, Medical College, Najran University, Najran 61441, Saudi Arabia; yealmalki@nu.edu.sa; 2 Department of Electronic Engineering, Quaid-e-Awam University of Engineering, Science and Technology, Larkana 76221, Pakistan; 3 Electrical Engineering Department, College of Engineering, Najran University, Najran 61441, Saudi Arabia; miditta@nu.edu.sa; 4 Department of Radiology, College of Medicine, Qassim University, Buraidah 52571, Saudi Arabia; 5 Eletrical Engineering Department, Sukkur IBA University, Sukkur 65200, Pakistan; ahmedali.shah@iba-suk.edu.pk

**Keywords:** breast cancer, mammogram images, image enhancement, image segmentations, K-means, pectoral muscle

## Abstract

Breast cancer is widespread worldwide and can be cured if diagnosed early. Using digital mammogram images and image processing with artificial intelligence can play an essential role in breast cancer diagnosis. As many computerized algorithms for breast cancer diagnosis have significant limitations, such as noise handling and varying or low contrast in the images, it can be difficult to segment the abnormal region. These challenges could be overcome by proposing a new pre-processing model, exploring its impact on the post-processing module, and testing it on an extensive database. In this research work, the three-step method is proposed and validated on large databases of mammography images. The first step corresponded to the database classification, followed by the second step, which removed the pectoral muscle from the mammogram image. The third stage utilized new image-enhancement techniques and a new segmentation module to detect abnormal regions in a well-enhanced image to diagnose breast cancer. The pre-and post-processing modules are based on novel image processing techniques. The proposed method was tested using data collected from different hospitals in the Qassim Health Cluster, Qassim Province, Saudi Arabia. This database contained the five categories in the Breast Imaging and Reporting and Data System and consisted of 2892 images; the proposed method is analyzed using the publicly available Mammographic Image Analysis Society database, which contained 322 images. The proposed method gives good contrast enhancement with peak-signal to noise ratio improvement of 3 dB. The proposed method provides an accuracy of approximately 92% on 2892 images of Qassim Health Cluster, Qassim Province, Saudi Arabia. The proposed method gives approximately 97% on the Mammographic Image Analysis Society database. The novelty of the proposed work is that it could work on all Breast Imaging and Reporting and Data System categories. The performance of the proposed method demonstrated its ability to improve the diagnostic performance of the computerized breast cancer detection method.

## 1. Introduction

Breast cancer is one of the most common diseases among women and it is considered a deadly type of cancer worldwide. According to the World Health Organization (WHO), 2.3 million women were diagnosed with breast cancer in 2020 and 685,000 deaths were reported worldwide due to breast cancer [1]. According to the 2018 report, the age-standardized rates (ASR) for incidence and mortality were 27.3 and 7.5 per 100,000 among Saudi women [2]. In a recent survey, it was shown that breast cancer is one of the nine leading causes of death among women in Saudi Arabia [3]. Cases of breast cancer are also on the rise in China, where it is becoming more common, with 12.2% of the female population in China currently living with the disease. Breast cancer is also responsible for 9.6% of deaths worldwide [4].

It is necessary to diagnose breast cancer as early as possible because the progression of the disease leads to serious complications and makes treatment more complex. Prompt diagnosis of disease may offer more treatment options, which could reduce the rising rate of breast cancer. Mammography images, and their analysis, are used for the diagnosis of breast cancer, in which they play a vital role besides being one of the processes used for disease diagnosis for prompt treatment of patients. However, the analysis of mammography images contains many challenging issues and it is challenging for a radiologist to diagnose and determine the site of the pathology [5,6]. Breast tissue density and the quality of the mammographic images are the two main factors that are used for analyzing mammographic images because the efficiency of mammography varies from 60 to 90%, malignancy when a biopsy is performed on a non-palpable mammographic abnormality usually ranges from 20% to 35% [7,8,9].

When radiologists analyze mammographic images, they are trying to find an abnormal region, and this may be easier once the images are clearer. Large amounts of calcification and asymmetric architectural distortion in the breast tissue are the most common findings in mammographic images [10]. The Breast Imaging Reporting and Data System (BI-RADS) is the mammographic standard, along with the quality assurance lexicon report published by the American College of Radiology (ACR). The main objective of the BI-RADS is to organize mammography reports between radiologists and standardize them for clinicians [11]. The BI-RADS classifies mammographic images into qualitative characteristics and characterizes their shapes and margins along with the mass density of the breast tissue. Radiologists classify mass in the BI-RADS according to defined characteristics, as explained in Table 1.

The biological structure of the breast is more important before the analysis of mammographic images. According to the biological structure of the breast, cancer cells spread from the lymph nodes and impact other parts of the body. The main cause of breast cancer is a dysfunction in the origin of the milk-producing duct, which is also known as invasive ductal carcinoma. Breast cancer can also develop in the glandular tissue of the breast and these are known as lobules. Research has proven that hormones, lifestyle and environmental changes also have an impact on the prevalence of breast cancer [12]. A low-dose breast X-ray is the standard process used to scan for disease. This process is known as mammography in biological terminology, and it has been proven to be the safest imaging modality for the cancer screening of the breast [6].

It is very important to implement the computerized technique for the early detection of breast cancer. One of the main factors before implementing a computerized breast cancer detection method is the analysis of mammography image databases. The implementation of a new algorithm would aid in the detection of breast cancer. Thorough analysis of the mammography image database for the implementation of a computerized breast cancer detection method can reduce the manual process of detecting the disease. This is a challenging issue for the analysis of a large number of mammographic image databases. The method of the image enhancement and segmentation of an abnormal region reduces the workload of medical experts and is necessary for studying the nature of the mammographic images before implementing any calculation algorithm for the detection of breast cancer [13].

In this research work, the three stages method is proposed of real-time database analysis for the detection of breast cancer. The first step was based on database classifications according to the BI-RADS and performed image processing steps with data uniformity. The second step was based on the removal of the pectoral muscle from the mammographic images. The third step was based on image enhancement to improve image quality and the segmentation of the abnormal region. In these three steps, the image processing techniques are used to obtain a well-enhanced and segmented abnormal region in order to be able to analyze the mammographic images for the purpose of diagnosing breast cancer. The main contributions of this research work are:

1. A classification method for large databases according to all levels in the BI-RADS.
2. The implementation of an image-enhancement technique for mammographic image pre-processing steps.
3. An image processing technique for pectoral muscle ablation.
4. The implementation of post-processing steps for the segmentation of an abnormal region.

The article is organized as follows. Section 2 summarizes the related work. Section 3 explains the proposed method in detail, along with each step of the proposed method. Section 4 deals with databases and measurement parameters. Section 5 reports the experimental results. The Section 6 contains discussion and future directions. Finally, the conclusion is presented in Section 7.

## 2. Related Work

Filtering techniques are used for the analysis of early-stage breast cancers. For example, the work by Mendez et al. [14] used spatial averaging filtering to smooth the image it was implemented on Histogram threshold techniques have also been used to detect abnormal regions. The new threshold baseline technique was proposed by Abdel et al. [15] for the removal of the pectoral muscle from an image. A multiresolution scheme based on the Hough transform was implemented by Karssemeijer and Brake [16] to remove the pectoral muscle from an image with precision. Their method was modified by Ferrari et al. [17], who based their work on certain image processing techniques for the removal of the pectoral muscle from an image. An adaptive histogram method was implemented by Raba et al. [18] to differentiate the cancerous region of the breast from the background image. These authors also used the region-growth-based method to remove the pectoral muscle from an image. A nonlinear diffusion algorithm was implemented by Mirzaalian et al. [19] for the removal of the pectoral muscle from an image. The Radon transform method was proposed by Kinsosita et al. [20] for the detection of abnormal cancerous regions. Later, the wavelet decomposition algorithm, using the tactics of background depth reduction in mammographic images, was used by Mario et al. [21] for cancer area detection; they achieved an accuracy of 85% using their method.

A new method was proposed by Wang et al. [22] for the automated detection of the pectoral muscle, which was based on a discrete-time Markov chain and an active contour model. A shape-based function with an average gradient was proposed by Chakraborty et al. [23] for the determination of the pectoral muscle boundary position as a straight line. The histogram-based thresholding method was implemented by Chen and Zwiggelaar [24] for breast cancer detection, and they used connected components to label the segmented binary image of the cancerous region. Next, they used the area based technique to remove the pectoral muscle and determine a starting point closer to the pectoral muscle boundary. The idea of using the triangular region-based method to isolate the pectoral muscle from the breast tissue was implemented by Maitra et al. [25], and they used the region growth technique to remove the pectoral muscle.

All of these methods used thresholding, region growing, or starting-point image techniques. These techniques did not give good performances because image uniformity was necessary; however, image contrast enhancement techniques have been used for decades to make video and image details more observable. A few contrast enhancement techniques are useful for mammographic images, but most conventional enhancement techniques are not useful for improving the contrast of mammographic images in order to impact the post-processing steps.

Over the past 21 years, many enhancement techniques have been implemented to improve low and variable contrast in mammographic images. Many detailed review articles are presented based on contrast enhancement techniques for mammographic images. Cheng et al. [26] discussed conventional methods for feature-based contrast enhancement techniques along with their advantages and disadvantages. Jianmin et al. [27] implemented an approach based on structure tensor and fuzzy enhancement operators for enhancing the contrast of mammographic images. Stojic et al. [28] implemented mammographic images based on local contrast enhancement and background noise suppression techniques. An improved method based on the histogram-based contrast enhancement technique for radiographic images was implemented by Ming et al. [29]. After reviewing all the methods for the early detection of breast cancer, it was observed that a new enhancement technique was needed for the pre-processing module for segmented breast cancer. This article contains the step-by-step analysis of mammographic images for the detection of a cancerous region of the breast. The proposed method contained the analysis of the hospitals database, a large database of around 2892 images, and provided the enhancement, as well as the detection of, the cancerous region of an image, as is explained in Section 3.

## 3. The Proposed Method

### 3.1. Data Classification

**Figure 1:**
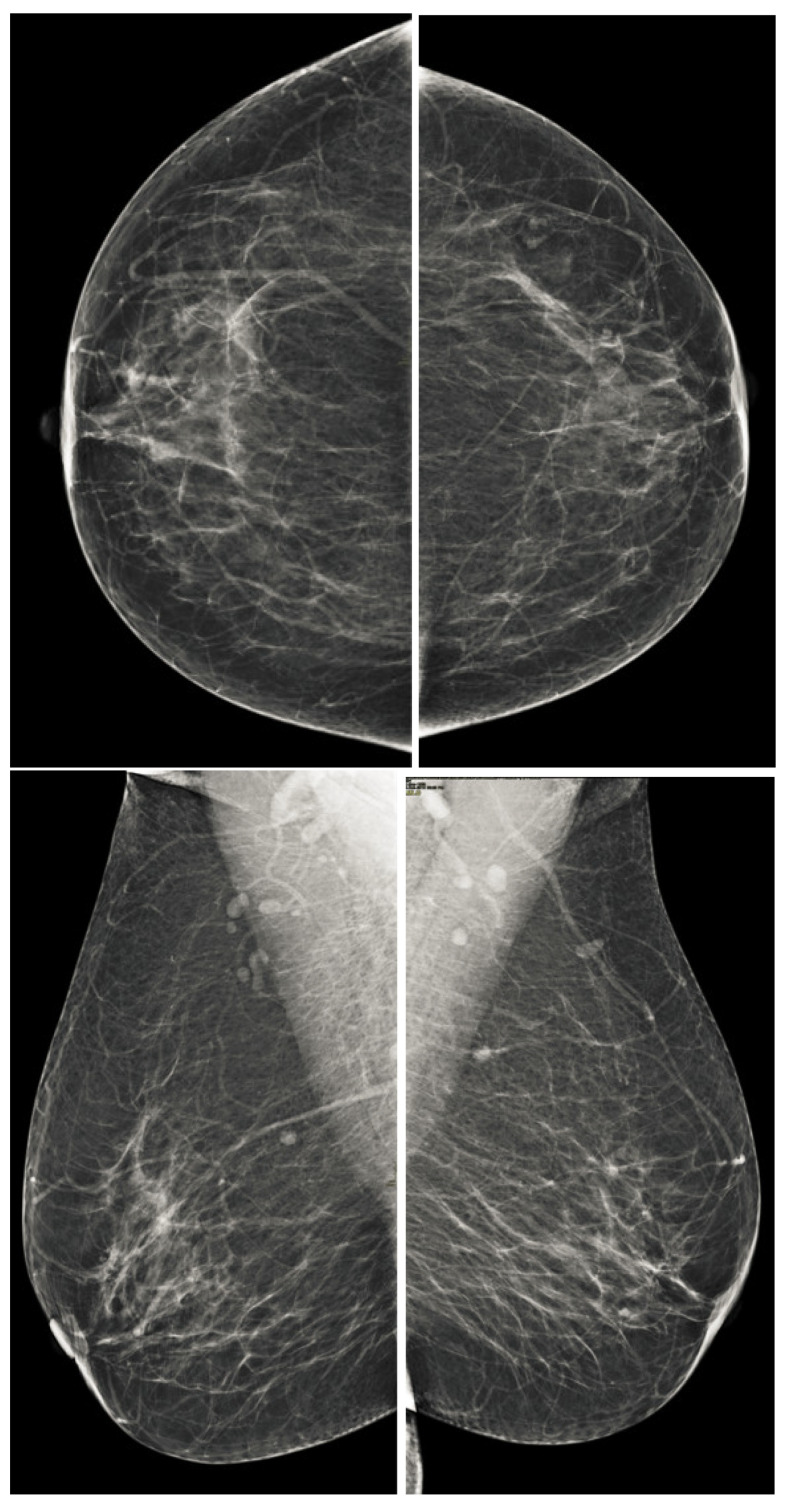
Representation of standard mammographic views. The first rows represent the CC view of the right and left breast, and the second row represents the MLO view of the right and left breast.

The implementation of any method depends on the nature of the data; it is necessary to analyze the databases before proposing a method of analysis for medical images. The mammographic images have analysed and found that there are two types of images: film screen mammographic images and digital mammographic images. The digital mammographic images are used for the proposed method. There were many challenges using digital mammographic images, and one of the most analyzed challenges was the detection and removal of pectoral muscles. The analysis of pectoral muscles depended on their geometric shape and their location in correspondence with their view [30,31]. There are two ways to view mammographic images, the carniocaudal (CC) view and the mediolateral oblique (MLO) view, and these views are shown in Figure 1. The pectoral muscle in the CC view is the semi-elliptical shape along the breast wall. The pectoral muscle in the MLO view is the shape in the upper mammogram coverage and roughly corresponds to the overlapping right-angled triangle shown in Figure 1. Both views have different issues and suffer from low contrast, but both contain the cancerous region in many cases. It is very important to remove the pectoral muscle from the breast region and classify the database of specific image views according to the BI-RADS classification as it facilitates the computerized process of detecting breast cancer.

The data is collected from different hospitals in the Qassim health cluster, Qassim province, Saudi Arabia. The database contained 2892 mammographic images and data categorized according to the Breast Imaging Data and Reporting System (BI-RADS). Detailed information on the number of images is given in Table 2. The 996 negative case images are analyzed of BI-RADS case to validate the pre-processing and post-processing steps.

**Figure 2:**
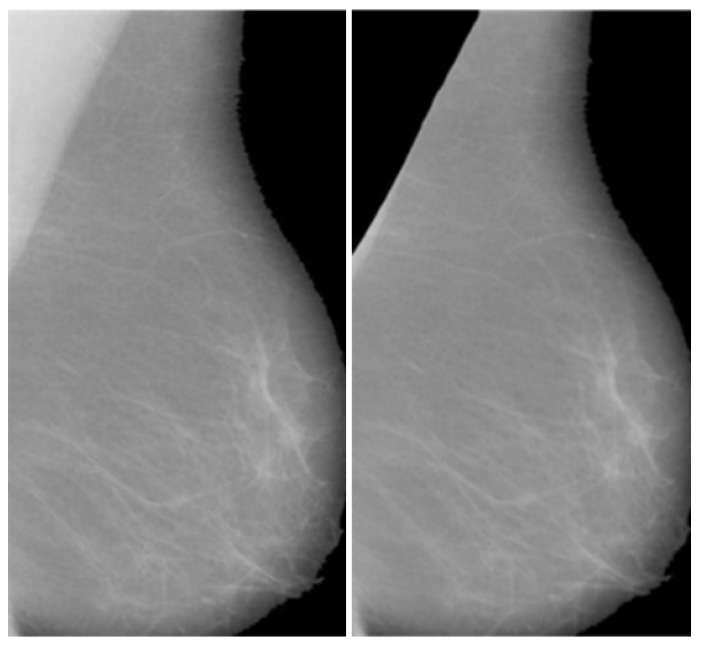
Final Breast image after pectoral muscle is partially removed.

After the classification of the database according to the BI-RADS, the next step was the removal of the pectoral muscles from the images; this was one of the novel steps that improve the efficiency of the overall method. The seed-based region growth technique is used to remove the pectoral muscles in order to obtain the mammary portions of the mammographic images; this area is the region of interest in mammographic images and contains the cancerous region. Seed region growth forms part of the image segmentation technique and contains two types: one type is pixel location value selection, and the other type is seed point selection. The seed point type is more precise and automatically gives an accurate selection based on the orientation of the image. In this proposed method, the starting point is used, which automatically selected the orientation of the mammographic images. The seed points were obtained by using the neighboring seed pixels and it was determined whether the required pixels should be added to or removed from the region. This process was continuous and iterative in nature until the segmentation of the breast region or removal of the pectoral muscle was achieved. The output of the pectoral muscle is removed in Figure 2 and provided an opportunity to analyze the breast area as well as to assist in the enhancement and segmentation techniques.

### 3.2. Pre-Processing Module: Image Enhancement

**Figure 3:**
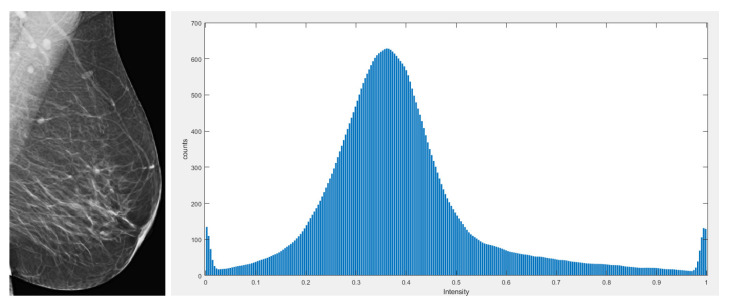
Enhanced breast image with its corresponding histogram.

There are many image-enhancement techniques for medical images and the suitability of each technique is determined by the nature of the medical image. Mammographic images contain varying amounts of contrast as well as noise. After removing the pectoral muscle, the morphological operations are used to evenly distribute the contrast. In the proposed method, the bottom and top hat operations are used to create uniform contrast in the images. These techniques perform well on mammographic images with a good pixel intensity distribution, as shown by the histogram with the enhanced image in Figure 3. These techniques helped the post-processing module. The mathematical representation of the morphological operations is illustrated in the equations below.

(1) $$T_{b}\left(f\right)=f•b-f.$$

Equation (1) represents the bottom hat operation and • depicts the close operation. The top hat operation was then applied to increase the contrast and control the change in contrast of the image. The mathematical representation of the top hat operation is defined in Equation (2). The symbol ∘ depicts the opening operation. An improved image was obtained with uneven illumination and noise suppression.

(2) $$T_{w}\left(f\right)=f-f\circ b.$$

### 3.3. Post-Processing Module

**Figure 4:**
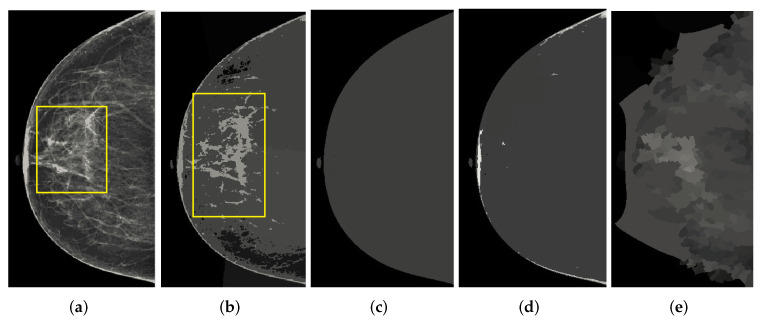
The output of our proposed post-processing step. The row shows output comparison of techniques: (**a**) Kmeans, (**b**) Kmeans Spatial, (**c**) Mean shift, (**d**) Mean shift Spatial and (**e**) Normalized cuts.

The segmentation of abnormal regions of the breast in mammographic images is a difficult task and many researchers are still working on it in order to implement new breast cancer detection methods. There are many segmentation algorithms for breast region detection, and some of the adaptive filtering techniques are used for abnormal region analysis. In this research work, K-means is used, K-means with spatial, mean shift, mean shift with spatial, and normalized cuts for the segmentation of mammograms. These are the most suitable methods for diagnostics of breast cancer from mammogram images. The normalized cut method was proposed by Shi and Malik [32] for use on natural images; its main purpose is to observe abnormal regions in mammographic images. This algorithm is based on graph theory. Normalized cuts work with image pixels as the node of a graph and treat segmentation as a graph partitioning problem. They calculate both the dissimilarity between groups as well as the total similarity within groups of image intensities. All these algorithms are based on clustering parameters, and K-means with a spatial range of 4 to 9 gave a better analysis of abnormalities on the mammographic images. The K-means method contained less noise compared to mean shift and mean shift with spatial, and normalized cuts gave more noise and also lost details. The output of these techniques is shown in Figure 4. It was clearly observed that the mean shift and normalized cuts techniques did not give a good segmentation of the mammographic images. However, K-means gave more detailed observations of the abnormal regions and the opportunity to analyze the databases. This was because every mammographic image database is different and K-means and K-means with spatial techniques, along with the proposed preprocessing module, can operate on databases. The effectiveness of this proposed method is explained in the results section.

## 4. Database and Measuring Parameters

### 4.1. Databases

The data is collected from different hospitals in the Qassim health cluster, Qassim province, Saudi Arabia, and this database contained 2892 mammogram images. The database was categorized according to the Breast Imaging Data and Reporting System (BI-RADS). Detailed information of the database is given in Table 2.

The Mammographic Image Analysis Society (MIAS) database [33] is also used. MIAS was created by British research groups with the aim of understanding images from mammograms, such as mammographic images from the UK National Breast Screening Programme. Images were digitized at 50 microns of pixel edge with a Joyce-Loebl scanning microdensitometer and a linear device in the 0–3.2 optical density range. Each pixel was represented by an 8-bit word. The MIAS database contained 322 digitized images of 161 pairs. The database had been reduced to a 200 micron pixel edge and padded so that all the images were 1024×1024.

### 4.2. Measuring Parameters

The proposed method was based on quantitative and visual observation. The following parameters were used to validate the proposed algorithm:

1. Peak signal to noise ratio (PSNR);
2. Effective measure of enhancement (EME);
3. Accuracy of an abnormal region.

The peak signal to noise Ratio (PSNR) and effective measure of enhancement (EME) parameters were used to validate the pre-processing module and its accuracy validated the post-processing module of the proposed method.

#### 4.2.1. Peak Signal to Noise Ratio (PSNR)

There are many processes for calculating PSNR; for PSNR, the value based on mean square estimation (MSE) is used. The first step is to calculate the MSE (as shown in Equation (4)) of the image tracks to calculate the PSNR (as shown in the equation). The PSNR is widely used to calculate the quality of images between the original image and the output image in terms of noise reduction. A large PSNR value means that the image has less noise compared to the original image.

(3) $$MSE=\frac{1}{MN}\sum_{m}{\sum_{n}\left|X\left(m,n\right)-\left.Y\left(m,n\right)\right|\right.}^{2}.$$

(4) $$PSNR=10\log_{10}\left[\frac{\left(L-1\right)}{MSE}\right].$$

where $X\left(m,n\right)$ and $X\left(m,n\right)$ are the grey level of input and output image at pixel position $\left(m,n\right)$, respectively. *L* is the maximum pixel value of the image, and it should be 255 in an 8-bit pixel image $\left[L=2^{n}-1=2^{8}-1=256-1=255\right]$. The PSNR is measured in decibels (dB).

#### 4.2.2. Effective Measure of Enhancement (EME)

The EME is a quantitative measure of image enhancement, and it gives information about the contrast of each image block. It is calculated using Equation (5), where $K_{1}$ and $K_{2}$ are the numbers of horizontal and vertical blocks in the enhanced image, respectively, and $I_{\max}\left(k,l\right)$ and $I_{\min}\left(k,l\right)$ are the image blocks maximum and minimum pixel values, respectively.

(5) $$EME=\frac{1}{K_{1}K_{2}}\sum_{L=1}^{K_{2}}\sum_{K=1}^{K_{1}}20\log\left(\frac{I_{\max}\left(k,l\right)}{I_{\min}\left(k,l\right)}\right).$$

### 4.3. Accuracy

The accuracy of a segmented abnormal region calculation contains all four measurements, including true positives (*TP*), true negatives (*TN*), false positives (*FP*), and false negatives (*FN*). These measurements are explained below.

(6) $$TP=\frac{\left(AS\cap AT\right)}{I}.$$

(7) $$TN=\frac{\left[\left(I-AS\right)\cap \left(I-AT\right)\right]}{I}.$$

(8) $$FP=\frac{\left[AS\cap \left(I-AT\right)\right]}{I}.$$

(9) $$FN=\frac{\left[\left(I-AS\right)\cap AT\right]}{I}.$$

The accuracy (*AC*) is the combinations of these parameters, as defined in Equation (10) below.

(10) $$AC=\frac{TP+TN}{TP+TF+FP+FN}.$$

## 5. Experimental Results Analysis

### 5.1. Analysis of Pre-Processing Module

The PSNR and the EME of each category of database are measured. Table 3 shows the performance of the proposed pre-processing steps. It can be seen that the proposed method improved the PSNR and EME. These pre-processing steps were known as image enhancement and the image enhancement technique is used with the post-processing steps for the detection of breast cancer.

### 5.2. Analysis of Post-Processing Module

**Figure 5:**
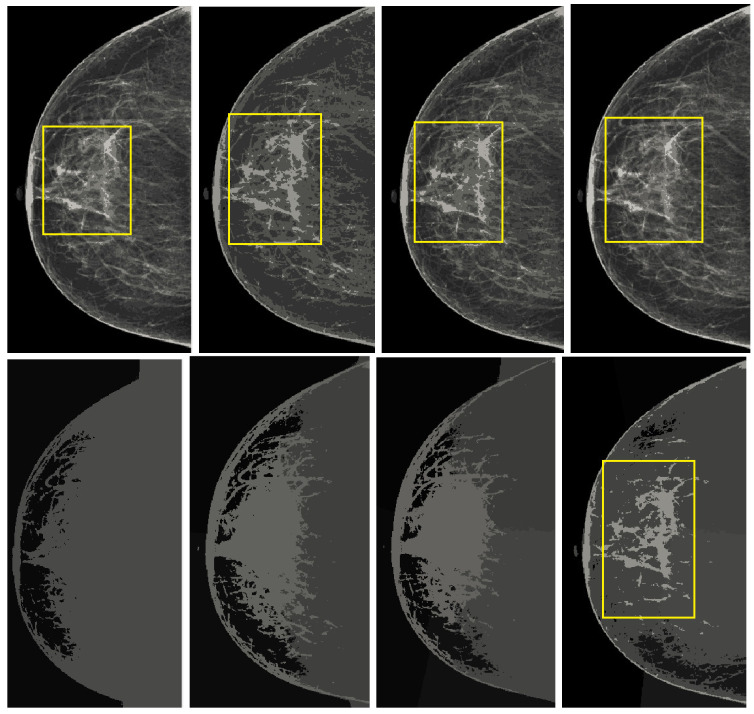
Comparison of output of K-means and K-means spatial. The first column shows output of K-means and K-means spatial with cluster 2. The second column shows output of K-means and K-means spatial with cluster 4. The third column shows output of K-means and K-means spatial with cluster 6. The fourth column shows output of K-means and K-means spatial with cluster 8.

The K-means and K-means with spatial in groups of 2 to 8 are analyzed. The best output of K-means is obtained from groups of 2, 4, 6, and 8, but K-means with spatial lost detail, as shown in Figure 5. It was clearly observed that an abnormal region can be easily seen in the K-means output with a yellow circle.

The accuracy of post-processing output is measured and shown in Table 4, and this yielded considerable accuracy, which showed the capability of the proposed method. The database created from the Qassim Health Cluster, Qassim Province, Saudi Arabia is also analyzed; however, the ability of the proposed method is measured by using databases from the Mammographic Image Analysis Society (MIAS) because many researchers have used these databases. The comparisons in Table 5 show that the proposed method performed comparably to other methods.

## 6. Discussion and Future Directions

Early detection of breast cancer can reduce the death rate. The rapid advancement of technology is needed to detect all types of abnormal regions with improved performance. The abnormal region in the mammogram can vary in size and shape, and the primary purpose is to segment the abnormal region with novel techniques. In this study, the computerized system is implemented to analyze mammography images for the detection of breast cancers. The various new methods are studied in the literature, and then the main problem is identified. There are three main challenges to implementing the computerized breast cancer detection method. The first step is based on database classification, the second is the implementation of pre-processing or image enhancement, and the third step is based on breast cancer segmentation.

In this proposed method, the first step was based on database classifications according to the BI-RADS and performed image processing steps with data uniformity. The second step was based on removing the pectoral muscle from the mammographic images. The third step was based on image enhancement to improve image quality and the segmentation of the abnormal region. In these three steps, the image processing techniques are used to obtain a well-enhanced and segmented abnormal area to analyze the mammographic images to diagnose breast cancer. These methodological contributions of this method were a classification method for large databases according to all levels in the BI-RADS, the development of an image-enhancement technique for mammographic image pre-processing steps, and an image processing technique for pectoral muscle ablation, the implementation of post-processing steps for the segmentation of an abnormal region.

The proposed method is validated on two databases: the Qassim health cluster, Qassim province, Saudi Arabia, and this database contained 2892 mammogram images. The database was categorized according to the Breast Imaging Data and Reporting System (BI-RADS) and The Mammographic Image Analysis Society (MIAS) database, which contained 322 images. The mammogram images have a size of 1024×1024, and abnormal regions have an average size of 128×128. It may vary in several cases. The proposed method gives good contrast enhancement with peak-signal to noise ratio improvement of 3dB. The proposed method provides an accuracy of approximately 92% on 2892 images of Qassim Health Cluster, Qassim Province, Saudi Arabia. The proposed method gives approximately 97% on the Mammographic Image Analysis Society database.

After analyzing the proposed method and comparing its performance with other existing methods, it can be seen that many researchers do not validate their methods on different databases because they have only validated their methods on the MIAS database. The comparability of the method can be analyzed to be validated on an extensive database. The proposed method in this research work is validated on extensive data from Qassim health cluster, Qassim Province, Saudi Arabia. The proposed method performed well on data from Qassim health cluster, Qassim Province, Saudi Arabia. We also validated the method on the MIAS database, and we used all images from MIAS because each image contained different challenges, and many researchers did not use all images as mentioned in the comparable Table 5. It can be observed that our method gives comparable performance compared to other methods. This comparative analysis indicates that this proposed method can be an aid tool for the radiologist for the analysis of mammography images for the early detection of the disease.

After a thorough analysis of mammography images, it is necessary to rely on a biologically theoretical study of mammography images, because there are many areas in mammography images necessary for analysis, such as the presence of abnormalities, the process of acquisition that has an impact on the observation of mammography images and the removal of the pectoral muscle. These factors are very important to manage and make the images more visible for the radiologist’s analysis for the diagnosis of disease progression and recommend early treatment. Based on the biologically theoretical concept of image mammography, this computerized method is implemented in this research work and the proposed methods performed well and performed comparable to existing methods.

This method can play an important role in the implementation in the shape of the product and it will be useful in hospitals, especially in the use of product in remote areas in the near future. These hardware models required computational mathematical techniques such as these studies [42,43,44] implemented for different medical images based on machine learning models as well as radio techniques for industrial use, as these studies are based on computational models based on mathematical techniques and machine learning. Our proposed method algorithm can be implemented in hardware based on more advanced machine learning techniques along with the necessary connections to equipment to automatically process patient data to the radiologist for mammography image analysis. In another future extension of this research work is the implementation of the proposed model in the form of mobile application for remote access to the patient to connect with the radiologist to analyze the images and obtain timely treatment.

## 7. Conclusions

In this research work, the proposed image processing techniques for the analysis of mammographic images from the Qassim Health Cluster, Saudi Arabia databases and proposed a complete model for the detection of breast cancer. The proposed model contained three stages: data classification based on the BI-RADS, the removal of the pectoral muscle, and the proposed image enhancement as pre- and post-processing modules for the detection of breast cancer. The good contrast is achieved in the images in proposed pre-processing steps and analyzed their impact on the post-processing module. The best segmented abnormal region is obtained. For more validation, the MIAS database is used and observed the performance of the proposed method. The proposed method is shown to have a comparable accuracy to other existing methods and an approximately 97% sensitivity and specificity. The novelty of the proposed work was that it could work on all the BI-RADS categories.

The proposed module, in terms of pre-processing and its impact on post-processing, gave a comparable performance to other methods, with a good image contrast in terms of visual perception and noise reduction, as well as better accuracy, sensitivity, and specificity. There is still room to improve the pre-and post-processing steps in order to obtain a more improved performance in the higher level of the BI-RADS. This image-enhancement technique could also be used to improve the performance of deep learning-based methods for breast cancer, as it could be used as an input technique for the training process. The good training of a database gives the best segmented output, and this is one of the ways that the proposed image-enhancement technique could improve the training process of the machine learning method. It could also improve the performance of traditional methods when used as a pre-processing module. 

## Figures and Tables

**Table 1:** Categorization of mass in the BI-RADS by radiologists.

Category	Remarks
0	Process is incomplete and requires further assessment.
1	Negative.
2	Benign finding.
3	Probably benign finding.
4	Suspicious abnormality.
5	Highly suggestive of malignancy.
6	Biopsy-proven malignancy.

**Table 2:** Database information.

Category	Number of Images
BI-RADs-1	996
BI-RADs-2	817
BI-RADs-3	371
BI-RADs-4	452
BI-RADs-5	256

**Table 3:** Performance of the proposed method based on the databases and categories.

	Without Pre-Processing Steps		With Pre-Processing Steps	
Category of BI-RADS	PSNR	EME	PSNR	EME
BI-RADS-1	28.13	5.12	30.18	7.36
BI-RADS-2	27.21	4.95	29.15	6.12
BI-RADS-3	26.05	4.31	29.01	6.12
BI-RADS-4	25.98	4.02	27.13	5.95
BI-RADS-5	25.54	3.98	26.97	4.97

**Table 4:** Performance of the proposed method based on the databases and categories.

Category of BI-RADS	Specificity	Sensitivity	Accuracy
BI-RADS-1	95.38	83.02	96.16
BI-RADS-2	94.98	81.04	94.23
BI-RADS-3	94.21	80.89	92.12
BI-RADS-4	94.08	80.03	91.13
BI-RADS-5	92.84	79.29	91.01

**Table 5:** Performance of the proposed method on MIAS.

Method	Images Used For Experiment	SP	SE	AC
Raba et al. [18]	320	-	-	98
Chen & Zwiggelaar [24]	322	-	-	92.8
Maitra et al. [25]	322	-	-	95.7
Peng et al. [32]	322	-	-	97.08
Wirth & Stapinski [34]	25	-	-	97
Kwok et al. [35]	322	-	88	83.9
Ferrari et al. [36]	84	-	-	96
Marti et al. [37]	65	-	-	97
Hu et al. [38]	170	-	91.3	-
Beena et al. [39]	60	-	-	83.33
Kaitouni et al. [40]	322	-	-	91.92
Podgornova et al. [41]	250	-	-	90.05
**Proposed Method**	**322**	**95.27**	**83.98**	**96.9**

## Data Availability

The data are available and can be shared upon request.
